# Reasons and Factors Affecting the Neonatal Intensive Care Unit (NICU) Length of Stay of Full-Term Newborns: A Systematic Review

**DOI:** 10.7759/cureus.73892

**Published:** 2024-11-18

**Authors:** Noha J Alhamawi, Hadeel A Alharbi, Mohammed H Alqahtani

**Affiliations:** 1 Pediatrics, King Fahad Medical City, Riyadh, SAU; 2 Neonatology, Al Yamamah Hospital, Riyadh, SAU

**Keywords:** hospital length of stay (los), intensive care unit, neonatal, risk factors, systematic review

## Abstract

There is a significant challenge in predicting the duration of the neonatal hospital length of stay (LOS) due to the complicated factors that affect newborns. Most studies are conducted among pre-term neonates, in which the gestational age is lower than 37 weeks and the birth weight of patients is considered a major risk factor for a prolonged LOS. No previous systematic review of the literature was conducted among the full-term population. This review aims to determine the risk factors for neonatal intensive care unit (NICU) LOS from multiple studies affecting full-term patients. The included studies focused on assessing reasons and factors affecting the NICU LOS of newborns, including "full-term newborns." We included studies that considered the NICU LOS as the primary outcome. Both retrospective and prospective studies were eligible for inclusion. The risk of bias for the included studies was evaluated using the Quality in Prognosis Studies (QUIPS) tool suggested by the Cochrane Prognosis Methods Group. The literature search of the databases identified 637 potentially relevant articles, among which 10 met the inclusion criteria and were selected for this review. Among the 10 studies, three risk factors were identified: disease-related, parent-related, and inherent factors. These factors constitute a critical risk factor most widely studied and consistently associated with LOS for the full-term population. Our findings offer an updated extensive summary of this aspect that has not been considered in detail in the literature. In conclusion, several critical risk factors affecting neonatal LOS were discovered in the published studies in this systematic review. However, there is a need for more prospective studies with standardized approaches that are crucial to confirm these findings and help develop effective interventions.

## Introduction and background

Neonates are considered a unique and highly sensitive patient population that requires special care. Over the last decades, advanced technology has significantly improved neonates' mortality and quality of life [1]. Newborn infants who require intensive medical care are usually admitted to a special unit in the hospital called the neonatal intensive care unit (NICU) [2]. Inborn admission rates to NICU vary significantly, from 1.1% to 37.7% of births [3]. Even though the majority of NICU admissions are for premature infants, there are a significant number of full-term newborns who also require NICU care. A cross-sectional analytical study conducted in Saudi Arabia found that the admission rate of full-term infants to the NICU during the study period was 4.1% in 2015 [4].

The number of infants who require intensive care has risen, with NICU admissions elevating since 2007, 6.4%, reaching 7.2% in 2018 [5]. The length of stay (LOS) of infants admitted to a neonatal unit can differ as infants born at term, near their due date, require special needs [6]. For instance, those who need cardiac surgery for a heart defect, are born very pre-term, require mechanical ventilation, or have other underlying diseases require a prolonged duration of hospitalization, as they usually require weeks to months of specialized neonatal intensive care [7].

Prolonged hospital stays may have several consequences for infants, families, and the entire healthcare sector. For instance, prolonged hospitalization puts infants at risk of neonatal complications due to concurrent exposure to external factors such as noise, bright light, and hospital-acquired infections (HAIs) [8,9]. According to some studies, these exposures may impact how HAIs evolve in the future, including candidemia [10], increase respiratory issues, such as transient tachypnea [11], and complicate the selection of antibiotics [12]. In addition, prolonged NICU LOS could also hinder the communication between parents and their infants and increase the cost of hospitalization [13,14]. Furthermore, prolonged hospital stays increase parents' anxiety and affect hospital resources [15].

There is a significant challenge in predicting the duration of hospital stay due to the complicated factors that affect newborns. Therefore, data about the LOS may assure parents and help clinical staff predict the duration according to the patient's case. Thus, to prevent prolonged NICU LOS, it is necessary to determine the risk factors that affect neonatal LOS.

In addition, most studies are conducted among the pre-term population, in which the gestational age is lower than 37 weeks and the birth weight of patients is considered a major risk factor for prolonged LOS [8,16-21]. A systematic review was conducted to assess the risk factors concerned with the pre-term population [22]. Still, no previous systematic literature review was conducted among the full-term population.

Therefore, assessing risk factors for NICU LOS is essential to accurately predict LOS among full-term patients, which is significant for adjusting hospital resources, counseling families, and effectively avoiding prolonged LOS [23,24]. Therefore, this review aimed to examine the potential risk factors for the NICU LOS of newborns from several studies affecting full-term patients.

## Review

Methodology

Search Strategy

A systematic literature search was conducted in PubMed, Scopus, MEDLINE, Ovid, and Cochrane Library for studies published in English from January 1988 to April 2024 by inserting keywords, Medical Subject Headings (MeSH), and combinations of these terms and synonyms. The search terms concentrated on "Intensive Care Units, Neonatal", "NICU", "Length of stay", "Stay Length", "Risk factors", "Causes", "reasons", "Influencing factors", Predictors, and their synonyms.

Inclusion Criteria

The included studies focused on assessing reasons and factors affecting the NICU LOS of newborns, including "full-term infants." We included studies that considered the NICU LOS as the primary outcome. Both retrospective and prospective studies were eligible for inclusion. Only studies published in English were retrieved for this review.

Exclusion Criteria

Studies were excluded for these reasons: (1) studies with no full access link, (2) studies with inappropriate objective/outcome, and (3) studies that included a pre-term population only where pre-term birth has been defined as any birth before 37 weeks [25], (4) study types such as case reports, letters, clinical trial studies, review articles, and systematic review articles, and (5) duplicate studies found in multiple databases or sources.

Data Extraction

Two reviewers extracted data on the outcomes of studies using a Microsoft Excel 2019 spreadsheet. The extraction process was conducted independently, in duplicate, and with a third senior reviewer, resolving disagreements when necessary. For each included study, data were collected on the following items: (1) basic information about the study, including first author, country, year of publication, study design, and aim, and (2) fundamental and important details about the research population, including exclusion criteria, population size, main risk factors affecting the NICU LOS of full-term newborns, and hospital LOS. Data was screened and reported following the Preferred Reporting Items for Systematic Reviews and Meta-Analyses (PRISMA) guidelines for systematic reviews [26].

Quality Assessment

The risk of bias for the included studies was assessed using the Quality in Prognosis Studies (QUIPS) tool recommended by the Cochrane Prognosis Methods Group [27]. An edited version of the QUIPS tool was implemented to review the quality of the study. This method of evaluating quality takes into account five potential bias categories as follows: (1) study population, (2) study attribution, (3) risk factor assessment, (4) outcome assessment, and (5) statistical analysis and reporting. The assessment of the study population contained five questions, and the remaining categories each contained three questions. The rating for the domains was assigned as low, moderate, or high risk of bias.

Results

**Figure 1:**
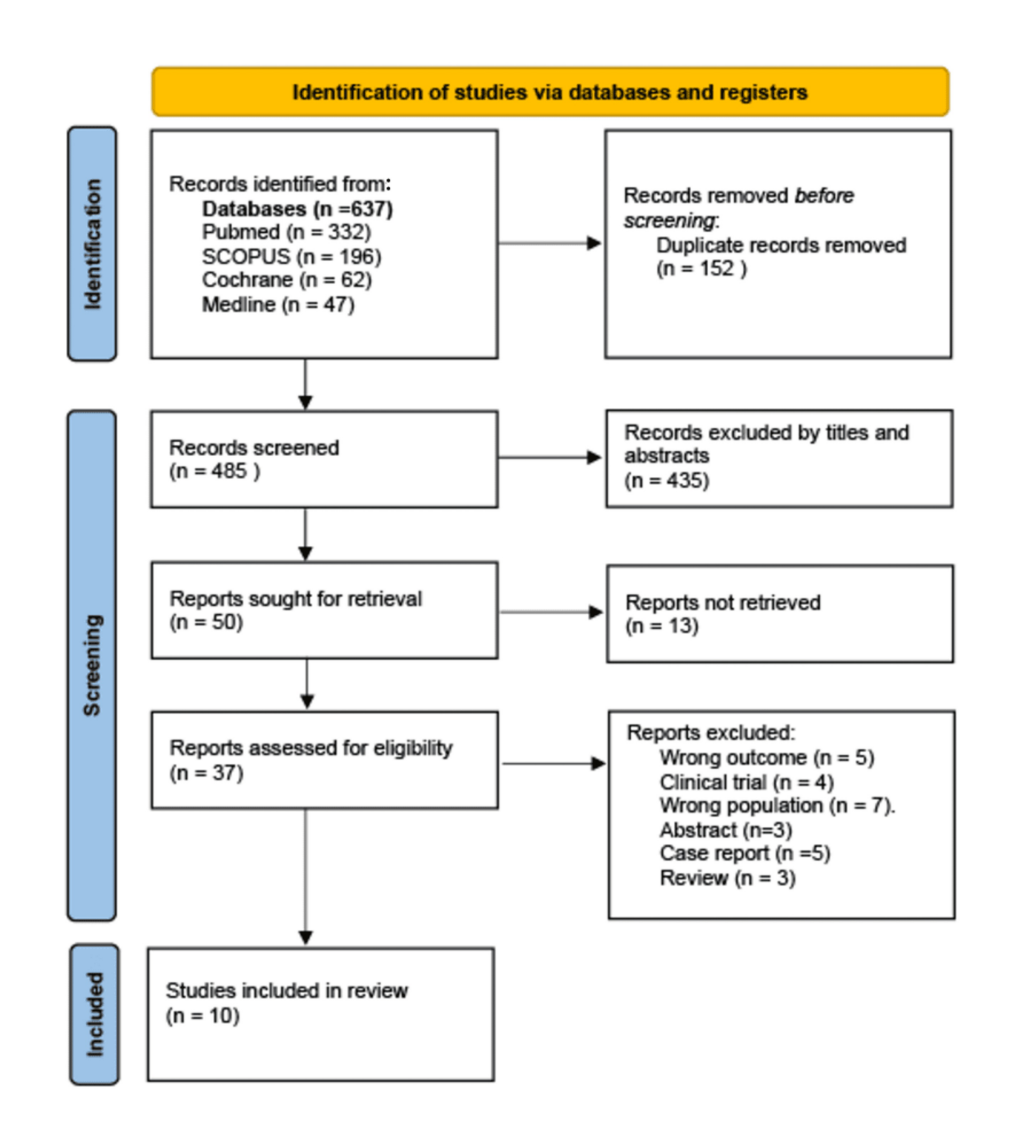
Flow diagram of study selection for the systematic review

Out of 637 potentially relevant articles, 152 duplicates were eliminated, with the remaining 485 articles to be screened for title and abstract. The full text of 50 articles was produced, of which 37 were assessed for eligibility. After the eligibility assessment, 10 articles met the inclusion criteria and were selected for inclusion in this review. The study identification and screening process is presented in Figure 1.

Overview of the Included Studies

The included studies were published between 2016 and 2024 in different countries across the world, including Turkey, China, India, Vietnam, the United Kingdom, and the United States. Out of these 10 studies, two were prospective studies [28,29], and the remaining eight studies were retrospective [30-37], of which one was a multicenter study [36] and nine were single-center studies [28-35,37]. The total population size was 30,122. Two studies with the largest population size included 16,094 newborns [32] and 5354 newborns [34]. The basic characteristics of the included literature are provided in Table 1.

**Table 1:** Characteristics of the included studies SD: standard deviation; IQR: interquartile range; DD: diaper dermatitis; HH: hyperinsulinemic hypoglycemia; NICU: neonatal intensive care unit; LRTI: lower respiratory tract infection; AKI: acute kidney injury; HAI: hospital-acquired infection; IV: intravenous; LOS: length of stay; ABR: antibiotic resistance; Gm: gram; GNB: gram-negative bacteria; RDS: respiratory distress syndrome; TTNB: transient tachypnea of the newborn; PIV: peripheral intravenous lines; RSV: respiratory syncytial virus; MAR: multiple antibiotic resistance; GIRmax: maximum glucose infusion rate; CPAP: continuous positive airway pressure

Author, year, country	Study design	Aim and outcome	Exclusions in the study	Study population	Population size	Hospital LOS	Factors associated with hospital LOS
Acar et al., 2024, Turkey [30]	Retrospective	Aims: to determine the risk factors by examining the sociodemographic characteristics of infants hospitalized in the NICU due to LRTI and to determine the underlying microbial factors and factors that affect the duration of hospitalization	NA	All gestations: birth weight: mean (SD)=3221±456 g. Gestational age: mean (SD)=38.2±1.5	All gestations: 57	≤7 days: mean (SD)=7.4±2.6 days	RSV positive (p=0.02). Oxygen therapy (p=0.03)
		Outcome: babies with a hospital stay of ≤7 days and >7 days. Compared for: demographics, presence and type of viral agent, and need for respiratory support		≤7 days: birth weight: mean (SD)=3119±415 g. Gestational age: mean (SD)=37.9±1.7 weeks. Hospitalization age: median=20.3 days	≤7 days: 33	>7 days: mean (SD)=8.2±3.3 days	
				>7 days: birth weight: mean (SD)=3361±481 g. Gestational age: mean (SD)=38.7±1.1 weeks. Hospitalization age: median=19.7 days	>7 days: 24		
Amodei et al., 2024, USA [31]	Retrospective	Aim: to explore the physical and sociodemographic predictors of infant LOS to determine whether any sociodemographic variables significantly contributed to the length of NICU hospitalization	NA	All gestations: birth weight: range=460-5273 g	All gestations: 757	≥1 day: mean (SD)=18.9±27.01 days. Range=1-217.5 days	Birth weight (lower). Ventilator use (day). Mean visits per day (lower)
		Outcome: babies with a hospital stay of ≥1 day (Group A) and >7 days (Group B). Compared for: infant demographics, maternal demographics, and ventilator usage		≥1 day birth weight: mean (SD)=2445.9±946.9 g	≥1 day: 757 of them, 409 stayed	>7 days: mean (SD)=32.3±30.9. Range=7-217.5 days	For >1 day (β=-0.342, p<0.001; β=0.654, p<0.001; β=-0.083, p<0.001, respectively)
				>7 days birth weight: mean (SD)=1999.8±799.2 g			For >7 days (β=-0.278, p<0.001; β=0.687, p<0.001; β=-0.086, p=0.001, respectively)
Xie et al., 2022, China [32]	Retrospective	Aim: to investigate the factors that influence the LOS in a NICU	Readmission. Remain in hospital for <1 day	All gestations (<28-42): weight range=1000-4000 g	16,094	Mean=11.08 days. Median=9.00 days. Range=1-141 days	(p<0.001). Gender (male). Patient source (inborn). Delivery method (C-section). Gestational age (lower). Birth weight (lower). Comorbidities (>1)
		Outcome: primary: LOS		N birth weight: g <1000=60, 1000-1499=707, 1500-2499=4542, 2500-3999=10, 155≥4000=630			
				Gestational age: weeks <28=79, 28-31+6d=923, 32-34+6d=2345, 35-36+6d=3255, 37-41+6d=9445≥42=47			
Esser et al., 2021, USA [33]	Retrospective	Aim: to examine the following: (1) the prevalence of DD in NICU; (2) the relationship of factors including clinical characteristics and DD; and (3) the contribution of DD and clinical characteristic factors on NICU LOS	(1) Transfer from an outside hospital after 24 hours. (2) Imperforate anus diagnosis or infants requiring diversion of stool to an ostomy for any portion of the NICU hospitalization. (3) Infants transferred out of NICU at any point during their stay. (4) Readmission to NICU	All gestations (22-42)	All gestations: 537	DD: mean (SD)=47.29±31.98 days	DD (no). Number of PIVs. Number of skin injuries. Number of days to full feeds. Race (White). Delivery type (vaginal). Gestational age (lower)
		Outcome: infant NICU LOS between DD and non-DD groups. Compared for: demographics, number of PIVs, number of skin injuries, and number of days to full feeds		DD birth weight: mean (SD)=1887.19±842.73 g. Gestational age: mean (SD)=32±3.61 weeks	DD: 180	Non-DD: mean (SD)=28.70±28.33 days	
				Non-DD birth weight: mean (SD)=2374.24±911.02 g. Gestational age: mean (SD)=34.47±3.89 weeks	Non-DD: 357		
Schoeneberg et al., 2021, USA [34]	Retrospective	Aim: to determine the risk factors for increased postoperative LOS in children with coarctation of the aorta	Associated cardiac defect, except atrial septal defect and patent ductus arteriosus. Dysfunctional aortic valve. Aortic insufficiency. Aortic stenosis. Underwent repair of the aortic valve	All gestations: hospitalization age: median (IQR)=6 (1-31) days	5354	Total LOS: mean (SD)=21.6±37.5 days	Mechanical ventilation. Gastrostomy. Prematurity/low birth weight (<37 weeks/≤2499 g). Large/small intestinal surgery. Tracheostomy. Congenital respiratory anomalies. Renal failure. Hospitalization day (lower)
		Outcome: all pediatrics <3 months from 2004 to 2018 underwent surgery. Primary: postoperative LOS. Secondary: in-hospital mortality. Compared for: demographics, comorbidities, genetic diagnoses, and procedures performed within the same hospitalization				Total ICU LOS: mean (SD)=8.8±18.0 days	
						Total NICU LOS (days): mean (SD)=22.3±38.1 days	
Singh et al., 2021, India [28]	Prospective	Aim: to develop a model for LOS prediction for each gestation category of a neonate using the most associated independent variables	Congenital anomalies. Palliative care. Discharge on request. Transfer death cases	All gestations: birth weight: >37 weeks: mean±SD=2737±537. Gestational age: >37 weeks: 38.5±1.0	Out of 836 patients, 273 were born after 37 weeks of gestation	>37 weeks: median (IQR)=4 (3) days	Gestational age. Nutrition orders (protein deviation). Medication orders (medication not required, dose deviation, and no deviation in medication dosage). Clinical diagnosis (severe RDS, RDS with TTNB, and sepsis)
		Outcome: to predict LOS based on risk factors, including perinatal and antenatal details, deviations in nutrition and medication dosage from guidelines, and clinical diagnoses encountered during NICU stay					
Peters et al., 2019, Vietnam [29]	Prospective	Aim: to assess the mortality and LOS attributable to ABR in GNB causing HAI in a Vietnamese NICU	Death within 24 hours after admission. Negative HAI cases	All gestations: birth weight: mean (SD)=1984.3±891 g. Median (range)=1800 (600-4500) g. Gestational age: mean (SD)=34±5.2 weeks. Median (range)=34 (24-40). Hospitalization age: mean (SD)=8±9.2 days. Median (range)=4 (0-44) days	327	Mean (SD)=38.7±26.0 days. Median=30 days	Birth weight (lower) (p<0.001). MAR score (higher) (p=0.029)
		Outcome: HAI-positive neonates are caused by MAR bacteria. Primary: LOS. Secondary: mortality. Through: demographics, time at risk, number of comorbidities, and number of invasive devices					
Kozen et al., 2018, UK [35]	Retrospective	Aim: to identify the perinatal factors and cost of care associated with transient neonatal HH	Birth weight of less than 1.8 kg. Gestational age of less than 34 completed weeks	All gestations: HH: birth weight: median (IQR)=3055 (2481-3524) g. Gestational age: median (IQR)=38.4 (37.1-39.5) weeks. Hospitalization age: median (IQR)=6 (4-14) hours	474	HH: median (IQR)=6 (3-12) days	Treatment with diazoxide with infants with HH (yes) (p<0.001). Insulin level (higher) GIR_max_ (higher)
		Outcome: the neonates were divided into two groups (HH and non-HH). Compared for: infant demographics, maternal factors, peripartum factors, and length and cost of stay		No HH: birth weight: median (IQR)=2855 (2450-3460) g. Gestational age: median (IQR)=38 (36.6-39.3) weeks. Hospitalization age: median (IQR)=9 (6-15) hours	HH: 40		
					Non-HH: 434	No HH: median (IQR)=3 (2-5) days	
Jetton et al., 2017, USA [36]	Retrospective	Aim: to establish whether neonatal AKI is independently associated with increased mortality and LOS in the NICU	Admission at age 14 days or older. Congenital heart disease requiring surgical repair within seven days of life. Lethal chromosomal anomaly. Death within 48 hours of admission. Inability to determine AKI status. Severe congenital kidney abnormalities	All gestations: N (%) no AKI: birth weight: ≤1000 g=112 (8%), 1001-1500 g=238 (17%), 1501-2500 g=552 (39%), ≥2501 g=513 (36%). Gestational age: 22-<29 weeks=142 (10%), 29-<36 weeks=748 (53%). ≥36 weeks=527 (37%)	2022	No AKI: median (IQR)=19 (9-36) days	AKI (yes) (p<0.001). Stages of AKI stages (higher)
		Outcome: the neonates were divided into two groups (AKI and non-AKI). Compared for: infant demographics, maternal factors, reason for admission, comorbidities, and LOS		AKI: birth weight: ≤1000 g=119 (20%), 1001-1500 g=57 (9%), 1501-2500 g=124 (21%), ≥2501 g=302 (50%). Gestational age: 22-<29 weeks=131 (22%), 29-<36 weeks=168 (28%), ≥36 weeks=306 (51%)	No AKI: 1417	AKI: median (IQR)=23 (10-61) days	
					AKI: 605		
Kurek Eken et al., 2016, Turkey [37]	Retrospective	Aim: to evaluate the maternal and neonatal risk factors associated with the LOS in the NICU	Deaths and transferred patients. Missing data on hospital LOS and maternal information	Birth weight: mean (SD)=2449.03±880.27 g. Gestational age: 23-43 weeks. Mean (SD)=35.04±3.64	3607	Mean (SD)=15.03±20.75 days. Range=1-257 days	(p<0.001). Gestational age (lower). Birth weight (lower). Maternal age (higher). Apgar score first min and fifth min (lower). Duration of sNIPPV (higher). Duration of CPAP (higher). Duration of nsNIPPV (higher)
		Outcome: to assess LOS. Through: demographics and obstetric risk factors					

Study Population

The studies included a range of infants with various gestational ages and birth weights, and full-term neonates were included in the study population. Exclusion criteria in the selected studies included missing data on hospital LOS and maternal information [37], readmissions [32,33], hospital stay <1 day [32], transfer to an outside hospital, imperforate anus [33], cardiac defects, aortic valve issues (dysfunctional aortic valve, aortic insufficiency, aortic stenosis, or repair of the aortic valve) [34], congenital anomalies, palliative care, discharge on request [28,33], death [28,29,36,37], birth weight <1.8 kg, gestational age <34 weeks [35], negative HAI cases [29], admission at age ≥14 days, congenital heart disease, surgical repair within seven days of life, fatal chromosomal anomaly, inability to determine acute kidney injury (AKI) status, and severe congenital kidney abnormalities [36] (see Table 1).

Risk Factors Affecting Hospital LOS

The 10 included studies described 39 statistically significant risk factors for NICU LOS. These variables are grouped into three broad categories: inherent factors including diseases and conditions of the newborn (38.5%, 15/39), infant and maternal demographics (30.75%, 12/39), and therapy, medication, and nutrition orders (30.75%, 12/39). Details of the risk factors extracted from each study are provided in Table 2.

**Table 2:** Risk factors for influencing NICU LOS included in the analysis of each study DD: diaper dermatitis; PIV: peripheral intravenous lines; RSV: respiratory syncytial virus; ABR: antibiotic resistance; HH: hyperinsulinemic hypoglycemia; AKI: acute kidney injury; RDS: respiratory distress syndrome; TTNB: transient tachypnea of the newborn; GIRmax: maximum glucose infusion rate; sNIPPV: synchronized non-invasive positive pressure ventilation; CPAP: continuous positive airway pressure; nsNIPPV: non-synchronized non-invasive positive pressure ventilation; NICU: neonatal intensive care unit; LOS: length of stay; MAR: multiple antibiotic resistance

Risk factors	Acar et al., 2024 [30]	Amodei et al., 2024 [31]	Xie et al., 2022 [32]	Esser et al., 2021 [33]	Schoeneberg et al., 2021 [34]	Singh et al., 2021 [28]	Peters et al., 2019 [29]	Kozen et al., 2018 [35]	Jetton et al., 2017 [36]	Kurek Eken et al., 2016 [37]
Birth weight	-	X	X	-	X	-	X	-	-	X
Gestational age	-	-	X	-	X	X	-	-	-	X
Maternal age	-	-	-	-	-	-	-	-	-	X
Male gender	-	-	X	-	-	-	-	-	-	-
Mean parents' visits per day	-	X	-	-	-	-	-	-	-	-
Ventilator use disease	-	X	-	-	X	-	-	-	-	-
DD	-	-	-	X	-	-	-	-	-	-
Number of PIV	-	-	-	X	-	-	-	-	-	-
RSV	X	-	-	-	-	-	-	-	-	-
Number of skin injuries	-	-	-	X	-	-	-	-	-	-
Number of days to full feeds	-	-	-	X	-	-	-	-	-	-
Congenital respiratory anomalies	-	-	-	-	X	-	-	-	-	-
Respiratory (tracheostomy)	-	-	-	-	X	-	-	-	-	-
Large/small intestine series	-	-	-	-	X	-	-	-	-	-
Clinical diagnosis (sepsis)	-	-	-	-	-	X	-	-	-	-
ABR	-	-	-	-	-	-	X	-	-	-
Severe RDS	-	-	-	-	-	-	-	-	-	-
RDS with TTNB	-	-	-	-	-	-	-	-	-	-
Neonatal HH	-	-	-	-	-	-	-	X	-	-
Treatment with diazoxide with infants with HH	-	-	-	-	-	-	-	X	-	-
GIRmax	-	-	-	-	-	-	-	X	-	-
AKI	-	-	-	-	X	-	-	-	X	-
Stages of AKI	-	-	-	-	-	-	-	-	X	-
Hospitalization day	-	-	-	-	X	-	-	-	-	-
Nutrition orders (protein deviation)	-	-	-	-	-	X	-	-	-	-
Medication orders	-	-	-	-	-	X	-	-	-	-
MAR score	-	-	-	-	-	-	X	-	-	-
Apgar score first min and fifth min	-	-	-	-	-	-	-	-	-	X
Duration of sNIPPV	-	-	-	-	-	-	-	-	-	X
Duration of CPAP	-	-	-	-	-	-	-	-	-	X
Duration of nsNIPPV	-	-	-	-	-	-	-	-	-	X

The risk factors that were commonly studied and most consistently related to NICU LOS included comorbidities, diaper dermatitis (DD), respiratory syncytial virus (RSV), number of days to full feeds, congenital respiratory anomalies, respiratory and abdominal surgeries, clinical diagnosis (sepsis), number of skin injuries, multiple antibiotic resistance (MAR) score, antibiotic resistance (ABR), severe respiratory distress syndrome (RDS), RDS with transient tachypnea of the newborn (TTNB), gastrostomy, large/small intestinal surgery, neonatal hyperbilirubinemia (NNH), tracheostomy, hyperinsulinemic hypoglycemia (HH), and treatment with diazoxide in infants with HH. Two studies considered some form of renal failure and AKI, three found that birth weight is a common risk factor among infants, and four concluded that gestational age affected the NICU LOS. Oxygen therapy, a lower Apgar score in the first min and fifth min, mechanical ventilation, duration of synchronized non-invasive positive pressure ventilation (sNIPPV), duration of continuous positive airway pressure (CPAP), duration of non-synchronized non-invasive positive pressure ventilation (nsNIPPV), number of peripheral intravenous lines (PIVs), medication not required, dose deviation, no deviation in medication dosage, energy deviation, nutrition orders, and protein deviation were also considered risk factors.

Quality of the Studies

Using the QUIPS tool, all studies were judged to be at low risk of bias in study attrition and outcome measurement domains as shown in Table 3. One study was judged to be at moderate risk of bias for statistical analysis and reporting due to the granularity of the datasets [34]. Other studies were judged to be at moderate risk of bias for risk factor measurement due to missing data in the methodology and a lack of standardized assessment tools or measurement [29,32-35,37]. One study was judged to be at high risk of bias [33], and three studies were judged to be at moderate risk of bias for the study population due to a lack of appropriate representative population size [28,34,36].

**Table 3:** Risk of bias assessment using the QUIPS tool QUIPS: Quality in Prognosis Studies

Author, year, and country	Study population	Study attribution	Risk factor measurement	Outcome measurement	Statistical analysis and reporting
Acar et al., 2024, Turkey [30]	Low	Low	High	Low	Low
Amodei et al., 2024, USA [31]	Low	Low	Low	Low	Low
Xie et al., 2022, China [32]	Low	Low	Moderate	Low	Low
Esser et al., 2021, USA [33]	High	Low	Moderate	Low	Low
Schoeneberg et al., 2021, USA [34]	Moderate	Low	Moderate	Low	Moderate
Singh et al., 2021, India [28]	Moderate	Low	Low	Low	Low
Peters et al., 2019, Vietnam [29]	Low	Low	Moderate	Low	Low
Kozen et al., 2018, UK [35]	Low	Low	Moderate	Low	Low
Jetton et al., 2017, USA [36]	Moderate	Low	Low	Low	Low
Kurek Eken et al., 2016, Turkey [37]	Low	Low	Moderate	Low	Low

Discussion

This is the first systematic review of the risk factors affecting NICU LOS, with eight studies focusing only on the full-term population. Among the 10 studies we included, three broad risk factors were identified: inherent factors including diseases and conditions of the newborn, infant and maternal demographics, and therapy, medication, and nutrition orders. These factors constitute a critical risk widely studied and consistently associated with the LOS for the full-term population. The findings of our systematic review offer a valuable, updated, extensive summary of this aspect that has yet to be considered in detail in the literature. This review will provide a new view that may guide the development of interventions to reduce the NICU hospital LOS.

Previous studies focused on the overall risk factors of neonates that contribute to prolonged LOS and on pre-term populations. A previous systematic review assessed the major risk factors that cause prolonged LOS cases due to low gestational age and other medical conditions of pre-term populations [22]. However, our research has shown that full-term infants have longer hospital stays if they have certain characteristics or conditions. For instance, full-term infants encounter longer hospital stays due to factors such as male gender, lower gestational age, lower birth weight (<37 weeks/≤2499 g), RSV-positive status, oxygen therapy, ventilator use, multiple comorbidities, more PIVs, and skin injuries. Extended stays are also linked to mechanical ventilation, gastrostomy, intestinal surgery, tracheostomy, congenital respiratory anomalies, severe RDS, TTNB, sepsis, high MAR scores, treatment with diazoxide in HH, high insulin levels, and higher maximum glucose infusion rate (GIRmax). Additionally, renal failure, AKI, and prolonged sNIPPV, CPAP, and nsNIPPV contribute to longer hospitalizations, as do higher maternal age and lower Apgar scores. In contrast, factors that reduce hospital LOS include higher mean visits per day, White race, vaginal delivery, absence of DD, and lower hospitalization days.

Among the included studies, Schoeneberg et al. found that the hospital LOS of neonates increases due to prematurity, low birth weight, and lower admission age [29,34]. Although many studies typically excluded cases involving congenital anomalies, Schoeneberg et al. and Singh et al. included them in their research. Their findings, which considered conditions, including RDS and RDS with TTNB, revealed a significant impact on neonatal LOS [28,34]. This could be due to the effects of these anomalies on patients' physiological functions, especially those requiring surgery. However, no extensive studies provided details about the relationship between LOS and neonates with congenital anomalies. Therefore, details about the types of anomalies should be defined in further research.

In addition, Xie et al., via a Cox regression analysis, revealed that male gender, born in the same admission hospital, C-section type of delivery, lower gestational age, lower birth weight, and more than one comorbidities were significantly associated with increased LOS [32]. Kurek Eken et al. also found that low gestational age, low birth weight, and lower Apgar score in the first min and fifth min were the most important confounders for the duration of hospitalization [37]. Additionally, a study by Esser et al. [33] found that more full feed days, numbers of PIVs and skin injuries, and no presence of DD played a crucial part as a risk factor for LOS. However, limited information collected about infant skin integrity hinders our understanding of DD in the NICU. Respiratory and abdominal surgeries were considered significant factors for neonates having coarctation of the aorta, which is a common congenital heart disease. The findings of this study could assist medical providers in the cardiac and NICU while counseling parents of infants about heart abnormalities [34]. Preventative measures should be improved to assess the relationship between LOS and DD development. Therefore, improved systems of gathering and analyzing DD data and its impact on the LOS are needed. However, it's uncertain if conditions affecting the heart or heart function better explain this correlation.

Furthermore, respiratory and abdominal surgeries and clinical diagnosis, including sepsis, ABR, HH, and AKI, were the disease factors contributing to prolonged LOS [29,36]. ABR in gram-negative bacteria was considered a significant risk factor for prolonged hospital stay in patients with HAI in the Vietnamese population. However, confounders such as comorbidities, severity of illness, and time at risk were essential to prevent biased results, which were rare practices in the field [29]. This is critical since neonates are a highly sensitive population that requires special care. In addition, ventilator use has a significant positive correlation with LOS, according to Amodei et al. [31]. Regarding AKI, the injury affects neonates of all gestational ages. Thus, future studies are needed to assess AKI systematically during high-risk events [36]. More studies are required to give data needed to support the development of methods designed to avoid AKI, and treatments to lower the burden of AKI, like renal support devices specialized for neonates, are needed to enhance the clinical outcomes of these vulnerable infants.

The optimal glucose infusion rate in infants differs according to gestational age and clinical condition, where GIRmax is associated with a higher LOS among patients with HH. A result of one of our included studies found that infants who did not need treatment had significantly shorter stays than infants treated with diazoxide. In infants with hyperinsulinism, the GIRmax was strongly associated with the cost of NICU admission and the total LOS [35]. Therefore, a proposed model for transferring to intermediate care after 60 days could help lower costs and enhance resource utilization.

Despite our valuable findings, this systematic review included several limitations. First, some studies were still missed even with the comprehensive search strategy. In addition, it was not possible to undergo a meta-analysis due to the significant heterogeneity in the study design, study population, definition of LOS, and statistical analysis methods. Finally, causal interpretations could not be made regarding the risk factors for LOS as most of the included studies were retrospective.

## Conclusions

This review found several important risk factors that affect newborns' hospital LOS. Full-term infants with certain characteristics or conditions, such as lower birth weight, RSV-positive status, and the need for oxygen therapy, tend to have longer hospital stays. Other factors, such as mechanical ventilation, gastrointestinal surgeries, and sepsis, contribute to extended stays. On the other hand, factors like higher mean visits per day, vaginal delivery, and absence of developmental delays are associated with shorter hospital stays. Identifying these factors is crucial for improving care and reducing hospital stays for full-term infants. Therefore, conducting more studies to target specific interventions for these risk factors is important to reduce the hospital LOS. Addressing risk factors with suitable interventions will help improve outcomes for full-term infants.
